# Case of Fallopian Pregnancy, with Rupture, Ending Fatally

**Published:** 1851-03

**Authors:** W. H. Watkins

**Affiliations:** Racine, Wisconsin


ART. IV.—*Case of Fallopian Pregnancy, wi'h Rupture, ending fatally.* Reported by W. H. Watkins, M. D., of Racine, Wisconsin.

MR. EDITOR:—The following unfortunate case having lately required my attention, I forward you the history.

I was called, at nine P. M., on the thirty-first of Dec., to attend Mrs. E—W—of this city, æt. thirty years.

Found her at her husband's jeweller's shop, some half mile from their residence. Mrs. W. was a strong, plethoric woman; health had been invariably good for years. To use her own expression, "had known what pain was only from what she had endured during confinement."

She was now suffering excruciating pain in the left Iliac fossa; pulse slow, weak, scarcely perceptible; hands and feet cold, and a deathlike pallor overspread the countenance; had vomited slightly; bowels regular: had eaten nothing more than usual; was nursing a child some eighteen months old; had menstruated regularly for the previous six months; discharge lessened in quantity, but slightly however, at the last two periods; time had returned for her being again unwell; had exerted herself considerably that afternoon, by lifting a stone out of a barrel, which weighed sixty to seventy pounds. In reply to my interrogatories concerning character of pain: she said it first resembled labor pain, but immediately became lancinating and changeable in its locality. Ordered Tinct. Opii, grs.

ʒi, and some gin being handy, gave one ounce in conjunction. This was repeated in twenty minutes; hot bricks were placed to her feet and warm cloths to abdomen. Being somewhat relieved, a sleigh was ordered, and I rode home with her. Pain had changed from left to right side—still severe. Gave Acet. Morphia gr. ½. An unsuccessful search for hernia was then made—ordered mustard cataplasm, large, to cover whole abdomen, and patient to be surrounded with warm woolen cloths. Morphia and gin sling repeated. Patient began to revive, pain decreased, and finally she fell into a gentle sleep fifteen minutes after eleven. I then returned to my office, leaving Morphine powders to be given with stimulus. Mr. W— called for me at one in the morning, saying pain had returned with increased violence. I stepped to the house of my partner, Dr. Hanchett, and engaged him to accompany me. We found our patient writhing in in agony; pain over region of the stomach; increased upon pressure; pulse hardly perceptible; skin cold, relaxed, but with only an appearance of moisture. She complained of thirst, dryness of fauces, &c.

Gave Acet. Morphia, gr. i.
Capsicum, gr. iii.
Camphor, gr. iii.

Fomentations, warm, to the abdomen. Warm drinks and warm gin sling to alleviate thirst. Anodynes and stimulants were frequently repeated. In searching for a cause for the above stated aggravating symptoms, poisoning, either accidentally or designedly, occurred to us. The tongue was pale, flabby and tremulous; the gums were pale around the edges. We learned that she had been painting her front room and also the window frames and sash throughout the house, and that the sabbath previous she had occasion to send some sugar of lead, which she had in the house, to a neighbor.

We came to the conclusion that she had been poisoned by lead, though such a supposition would not account for all the symptoms in attendance, and only an idiosyncracy would explain the severity of any. We left her more quiet at four in the morning, with directions to attendants to continue above treatment. Saw her at nine o'clock and found her in perfect state of collapse; learned upon inquiry that the opiates and stimulants had not been given, vomiting having supervened soon after our departure. Motion of the bowels had taken place, and I was informed by the old ladies that a show was discoverable.

Ordered, Brandy f℥iv.
Tinct. Capsici gr. xx.
Acet. Morphia gr. i.

and an enema of starch and laudanum.

Dr. Hanchett was again called in and we gave the stimulus in conjunction with the Ext. Belladonna gr. i frequently repeated.

Pain continuing, and patient not being relieved, further counsel was called. Drs. Groves, Page and Wooster saw her. Same treatment continued but without relief. And finally my patient succumbed a little past noon, only fifteen hours from time of attack.

A request for post mortem being acceded to, it was performed at ten A. M. the following day. There were present Drs. Hoy, Thompson, Talcott, Wilson, Wooster, Hanchett, and McNeill of Peoria, Illinois. Abdomen much distended. Upon making a vertical incision through parietes of the abdomen, we found the whole peritoneal cavity filled with bloody serum and large clots of coagulated blood—some five lbs. in all. The fallopian tube of the left side was enlarged, and a rupture, nearly round and only two lines in diameter, was discovered, and through it was seen a pale, fibrinous body. Upon opening it we found a fœtus, membranes, cord, &c., all perfect. The placenta presented at the rupture, being, in fact, a case of placental presentation.

The fœtus, judging from perfectness of development, rather than size, must have been from three to four months old. It was an inch long, head large, lower extremities well developed, membrana pupillaris quite distinct, mouth large and open, sex very evident, being a male.

Uterus double common size, lined with deciduous membrane one third inch thick. No connection between uterus and cavity in fallopian tube. The mouth of uterus not closed by lymph.

The principal point of interest in this case was in the diagnosis. In this there was a great mistake. Had there been an examination made by physical signs, doubtless a more satisfactory conclusion might have been arrived at; but no such exploration was made after I saw her the first time, and then there was only a slight tympanites.

It is of some interest as furnishing a case of tubal pregnancy where it was not supposed that the subject was pregnant.

Conjecture is the only resource left us as to the cause for such abnormal deviations from the prescribed course of nature.

The suggestion may be worth consideration however—whether nursing longer than nature dictates to be necessary, might produce such an effect. This idea is supported by the history of nearly all cases on record so far as I have had the privilege of examining.

WM. H. WATKINS, M. D.

RACINE, WISCONSIN, Jan., 1851.

*Remarks on the foregoing Case.*—In a note accompanying the foregoing article, the reporter requests us to add some remarks upon the case, as respects particularly, the diagnosis. Several circumstances combined to render the character of the attack obscure. The existence of pregnancy had not been suspected, and the continuance of lactation, together with the regular recurrence of menstruation, was opposed to such a suspicion, even had it been entertained. It is somewhat singular that the patient had not been aware of the presence of a tumor in the abdomen, but it does not appear from the history that any such discovery had been made. The symptoms of the outset denoted peritonitis, which was occasioned by the sudden rupture of the fallopian tube at that time, but it may readily be conceived that circumstances did not at once appear to justify the diagnosis of that affection; still less that the attack proceeded from such an origin. The case, however, may serve to illustrate the importance of a close consideration of symptoms and signs with reference to the discrimination of peritoneal inflammation from a neuropathic affection. Subsequently, on examining for hernia, it might be supposed perhaps that either the fallopian tumor would have been apparent, or a degree and kind of tenderness over the abdomen, which would have led to the supposition that peritonitis was present, if not to a conjecture as to the cause. The corpulency of the patient doubtless stood in the way of a successful exploration as respects the tumor, and owing to the great loss of blood with the consequent collapse, probably, the sensibility was so much obtunded that the abdominal tenderness was not so prominent as it otherwise would have been. Moreover, what tenderness was present seems to have been more marked in the epigastric region than elsewhere. Had the peritonitis been diagnosed it might under the circumstances have been attributed to other causes than rupture of the impregnated fallopian tube. Perforation of the intestine would have occasioned a train of symptoms in many respects similar, but less speedily followed by collapse and death, because there would have been less hæmorrhage. The latter was doubtless the immediate cause of death in this case.

A correct diagnosis, however, had it been made early, would have, in such a case, but little, if any influence on the treatment. The accident must almost, of necessity, prove fatal, and the only therapeutical indications were those which the symptoms rendered obvious, and which were adopted.

The history of the case is highly instructive, and should serve to impress the importance of bearing in mind the occurrence of fallopian pregnancy and rupture, as one of the causes of peritonitis suddenly developed.

